# Disease burden of myasthenia gravis in relation to oral corticosteroid dose: an insurance claims database study in Japan

**DOI:** 10.3389/fneur.2025.1662173

**Published:** 2025-10-22

**Authors:** Makoto Samukawa, Shingo Konno, Akiyuki Uzawa, Kentaro Taki, Hiroshi Todaka, Izumi Mishiro, Céline Quelen, Zuzanna Łukowicz, Renata Majewska, Yohei Ohashi

**Affiliations:** ^1^Department of Neurology, Kindai University Faculty of Medicine, Osaka, Japan; ^2^Department of Neurology, Toho University Ohashi Medical Center, Tokyo, Japan; ^3^Department of Neurology, Graduate School of Medicine, Chiba University, Chiba, Japan; ^4^Medical Affairs Rare Disease, UCB, Tokyo, Japan; ^5^Real World Evidence, UCB, Tokyo, Japan; ^6^Putnam, London, United Kingdom; ^7^Putnam, Kraków, Poland

**Keywords:** myasthenia gravis, corticosteroids, osteoporotic fracture, diabetes, cost

## Abstract

**Introduction:**

Oral corticosteroids (OCS) are used for maintenance treatment of myasthenia gravis (MG). Prolonged use of higher-dose OCS may provoke serious adverse events. Therefore, Japanese clinical guidelines recommend an OCS dose target of ≤5 mg/day. This retrospective study aimed to compare the burden of MG between patients achieving this target and non-achievers.

**Methods:**

Data were obtained from three Japanese healthcare databases (JMDC, NHI and LSEHS) between 2014 and 2021. Patients with MG starting immunotherapy were enrolled and data were collected over 2 years following start of immunotherapy. Exposure to OCS was determined from medication delivery records; achievers and non-achievers of the ≤5 mg/day target during follow-up were identified. Outcomes evaluated were confirmed incident diabetes, new osteoporotic fracture, and total and MG-related costs.

**Results:**

Overall, 459 patients were analyzed. Of these, 94 patients (58.4%) in the JMDC population, 96 (64.0%) in the NHI population and 119 (80.4%) in the LSEHS population achieved the ≤5 mg/day target. Incident confirmed diabetes in the JMDC population and new osteoporotic fractures in the LSEHS population were less frequent in target achievers than in non-achievers (*p* = 0.01 and *p* < 0.05, respectively). In target achievers in the JMDC and LSEHS populations, total and MG-related costs were lower (both *p* ≤ 0.01) than in non-achievers.

**Discussion:**

OCS dose target non-achievers carry a higher burden than achievers. Broader implementation of effective treatment strategies is required to reduce long-term use of higher-dose OCS and the associated burden.

## 1 Introduction

Myasthenia gravis (MG) is a rare autoimmune disease caused by pathogenic IgG autoantibodies and complement activation that disrupt the structure of the neuromuscular junction and impair synaptic transmission (1). The principal clinical manifestations are muscle weakness and abnormal muscular fatigue in response to exertion (2). The disease course is characterized by periods of disease stability with few clinical manifestations, punctuated by exacerbations requiring hospitalization. In the case of severe impairment of the respiratory muscles, these may be life-threatening (myasthenic crises) and require ventilatory support in an intensive care unit (3).

Treatment involves symptom management with acetylcholinesterase inhibitors (AChEI) and use of immunosuppressants to attenuate the underlying autoimmune disease process (4, 5). For MG exacerbations or crises, intravenous immunoglobulins (IVIg) or plasma exchange (PLEX) may be needed to eliminate autoantibodies. Many patients with MG can achieve a satisfactory quality of life (QoL) when treated sufficiently to control their symptoms (2, 6). However, this often involves using higher-dose oral corticosteroids (OCS). In Japan, the standard treatment of MG has historically involved the use of higher-dose OCS. However, long-term higher-dose OCS use carries an increased risk of potentially serious adverse events, including osteoporotic fractures and diabetes, which are the most common (7–14). In 2014, Japanese clinical practice guidelines were published, which introduced a major change in the treatment paradigm aimed at reducing long-term exposure to higher-dose OCS (15, 16). The overall treatment target in the 2014 Japanese guidelines was to achieve minimal manifestations (MM) of disease (17, 18) with an OCS dose of less than 5 mg/day prednisolone equivalents as rapidly as possible. The recommended strategy to achieve this target is to initiate an early fast-acting treatment (EFT), aggressive use of intravenous methylprednisolone (IVMP), plasmapheresis, IVIg or a combination of these (19). In addition to the 2014 national guidelines, more recently approved therapies for MG in Japan, such as eculizumab and zilucoplan, have demonstrated steroid-sparing effects (20, 21).

We recently performed an epidemiological study of OCS use by patients with MG in the real-world treatment setting in Japan using data from three health insurance claims databases (22). We found that use of OCS at a dose >5 mg/day over long periods of time remains high in everyday clinical practice 10 years after the publication of Japanese practice guidelines aimed at reducing exposure to OCS.

In the present study, we sought to highlight the burden of MG, such as the side effects of higher-dose OCS, in the three populations of patients with MG from the previous study, as a function of OCS use. The objective of the study was to estimate the incidence of OCS-related complications (confirmed diabetes and osteoporotic fracture). The outcomes were compared between patients who achieved and those who did not achieve the target of ≤5 mg/day OCS within 2 years after first diagnosis of MG.

## 2 Methods

### 2.1 Study design

This was a retrospective study performed in three Japanese health insurance claims databases, namely the Japan Medical Data Center (JMDC) Claims Database, the National Health Insurance (NHI) database and the Late-Stage Elderly Health Insurance (LSEHS) database. A cross-sectional design was used, which has been described in detail previously (22) and is summarized below. Patients with a confirmed first diagnosis of MG during the selection period were eligible. For the JMDC database, the selection period lasted from 1st January 2015 to 31st December 2019. For the NHI and LSEHS, the selection period lasted from 1st October 2014 until 31st December 2019.

The index date was defined as the date of the first dispensing of immunotherapies following the first documented claim with an International Statistical Classification of Diseases 10th Edition (ICD-10) diagnosis code for MG (G70.0) during the selection period. The immunotherapies of interest in this study were OCS, IVMP, PLEX/PP, IVIg with or without IVMP, CNIs, methotrexate, azathioprine, mycophenolate mofetil or eculizumab.

**Figure 1:**
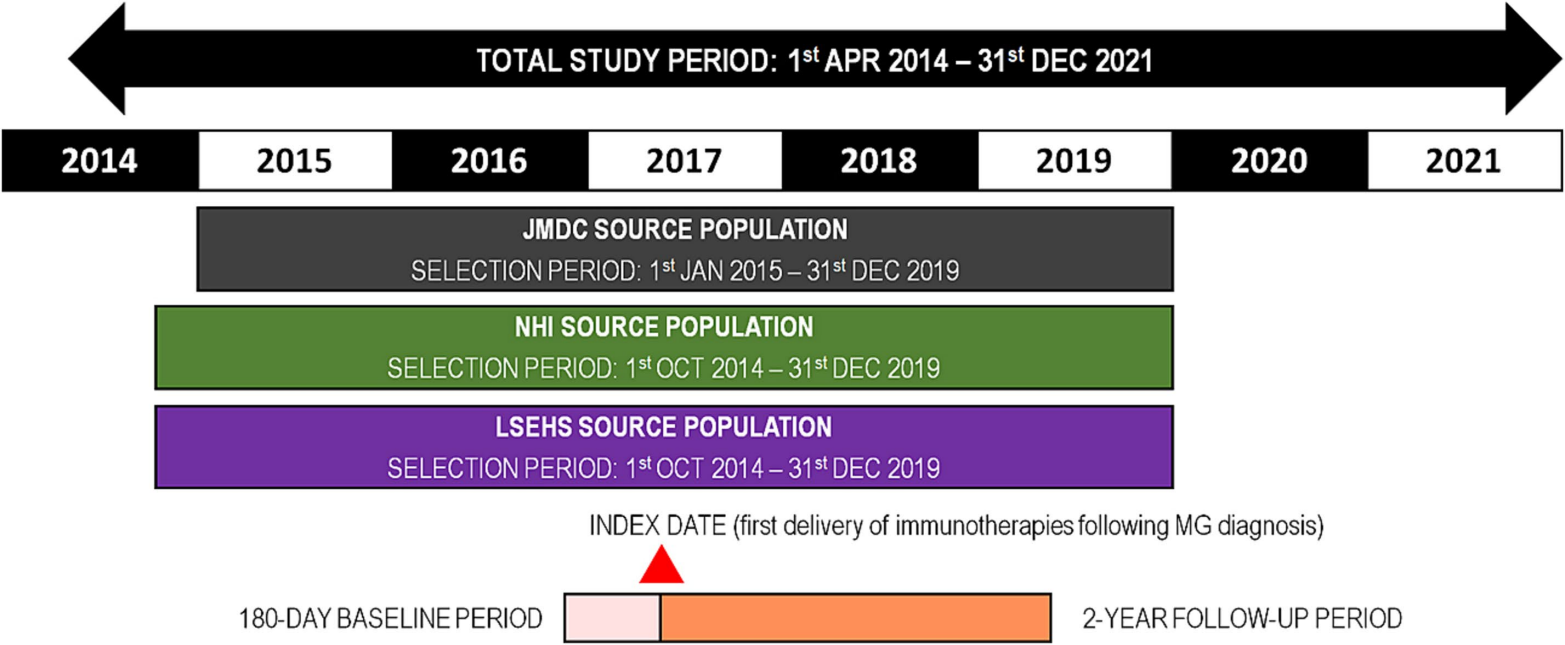
Study design. MG, myasthenia gravis; NHI, national health insurance; LSEHS, late-stage elderly health insurance; OCS, oral corticosteroid.

During a baseline period of 180 days before the index date, relevant medical history was documented. Patients were followed up from the index date for 2 years, or until the end of the study (31st December 2021), the end of insurance enrolment, or death (whichever occurred first). The total study period thus lasted from 1st April 2014 until 31st December 2021. The study design is illustrated in Figure 1.

### 2.2 Data sources

Data was retrieved from three Japanese health insurance claims databases, which are summarized below. Further information can be found in the Inventory of Japanese databases for clinical- and pharmaco-epidemiology research (23).

#### 2.2.1 JMDC claims database

The JMDC is a large claims database covering social health insurance of salaried workers and their dependents aged <75 years old provided by JMDC Inc. It contains inpatient, outpatient, and pharmacy claims of around 19 million cumulative beneficiaries in Japan since 2005. The database includes longitudinal, anonymized data on disease class, identified from ICD-10 disease codes, and medication prescription, classified by anatomical therapeutic chemical (ATC) class and medical procedures. The database contains information from annual health check-ups for certain beneficiaries, which includes height and weight.

#### 2.2.2 NHI database

The NHI database is part of the DeSC database. It contains information on around 15% of all the beneficiaries of National Health Insurance, which covers individuals younger than 75 years old who are unemployed, self-employed or retired, and their dependents. The database includes similar information to the JMDC database.

#### 2.2.3 LSEHS database

This database is part of the DeSC database and contains similar information to the NHI database. The LSEHS contains data specifically on individuals aged ≥75 years covered by the Advanced Elderly Medical Service System for elderly people provided by the Japanese government. It also includes individuals aged ≥65 years with significant disabilities. Data on around 17% of all LSEHS beneficiaries in Japan are available.

### 2.3 Patients

Eligible patients with MG were identified from the ICD-10 code G70.0 associated with any reimbursement claim. The inclusion criteria were documentation of a confirmed diagnosis of MG during the selection period, together with a documented MG-related serological test (antibodies directed against the acetylcholine receptor (AChR) or muscle-specific kinase (MuSK)) during the baseline period or at the index date, and a delivery of immunotherapies in the 90 days after diagnosis of MG. In addition, patients were required to be aged ≥16 years at the index date, and to be present in the database throughout the 180-day baseline period. The exclusion criteria were a claim for immunotherapies covering a period of >90 days any time before the index date, a claim for any MG treatment (medication, radiation therapy for thymoma or thymectomy) any time before the index date, or the absence of a visit with a MG diagnosis claim in the 6 months after the index date (22).

For the present study, the analysis was restricted to patients who had been prescribed an initial OCS treatment at the index date or in the following 90 days and who had achieved ≥2 years’ follow-up. The eligibility criteria for the present analysis are illustrated in Figure 1.

The study population was divided into two subgroups according to whether the patient had achieved a daily dose of ≤5 mg/day OCS during the two-year follow-up period or not. Achievement of ≤5 mg/day OCS was defined as ≥90 days of consecutive daily doses of OCS ≤ 5 mg (referred to as the maintenance period) with no gap >60 days between two consecutive prescription claims for OCS (referred to as the grace period). Patients who discontinued OCS (i.e., with a gap >60 days between two consecutive prescription claims) were considered to have achieved ≤5 mg/day OCS.

### 2.4 Exposure to oral corticosteroids

Exposure to OCS was calculated as the estimated daily dose from the number of tablets delivered and the period covered by the prescription. The daily dose was converted into prednisolone dose equivalents using the equivalence table proposed by Asare (24) and classified into low dose OCS (≤5 mg/ day) and moderate-to-high dose OCS (>5 mg/day).

### 2.5 Study variables

At the index date, age and gender were documented. Comorbidities of interest were diabetes mellitus and osteoporotic fracture, occurring any time before the index date or during the two-year follow-up period. These were identified from ICD-10 codes for hospitalizations or medication codes for specific treatments using previously described and validated search strategies for Japanese claims databases without any modification (25, 26). In addition, comorbidities contributing to the Charlson Comorbidity Index (CCI) (27) were also documented and the CCI calculated therefrom.

Costs of all claims documented in the databases during the six-month baseline period and the two-year follow up period were compiled and are presented as annualized costs in Japanese Yen (¥). A subset of MG-related costs was also identified, corresponding to all claims associated with an MG ICD-10 diagnosis code or a medication code for an MG treatment (OCS, IVMP, IVIg, AChEI, CNI, methotrexate, azathioprine, mycophenolate mofetil, eculizumab) or a procedure code for PP/PLEX, thymectomy or radiation therapy for thymoma.

### 2.6 Statistical analysis

Categorical variables are presented as frequency counts, and percentages and continuous variables as mean values with their standard deviations (SD) or median values with their interquartile range [IQR] as appropriate. Categorical variables were compared between patients who achieved the ≤5 mg/day OCS target versus those who did not with the χ^2^ test or Fisher’s exact test as appropriate. Continuous variables were compared with Student’s *t*-test or the Kruskal-Wallis test as appropriate. Statistical significance was taken as a probability threshold of *α* = 0.05.

Logistic regression models were implemented for each of the three populations in order to evaluate the association between the incidence of confirmed diabetes and osteoporotic fracture in the first 2 years of follow-up on the one hand and achieve the target of ≤5 mg/day OCS during the same period on the other. Certain variables considered to be particularly relevant were introduced as forced variables, including age, gender, hospital size (≥500 beds versus <500 beds), number of hospital visits for MG in the first 6 months of follow-up and, in the case of osteoporotic fractures only, diagnosis of osteoporosis. Patients with confirmed diabetes before the index date were excluded from the logistic regression analysis of confirmed incident diabetes. Data are presented as odds ratios (OR) with their 95% confidence intervals (CI).

## 3 Results

### 3.1 Study population

**Figure 2:**
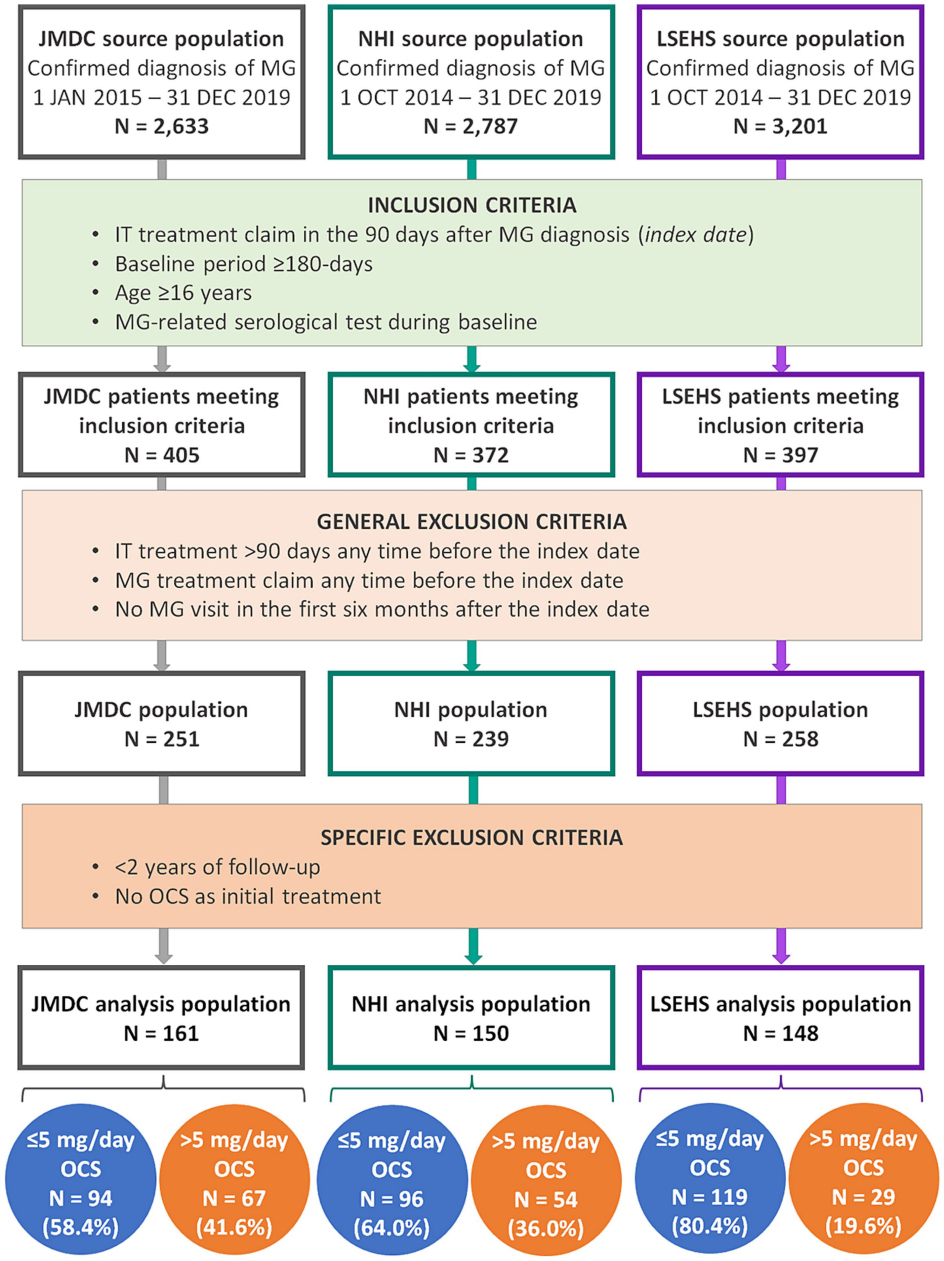
Patient flow diagram. MG, myasthenia gravis; IT, immunotherapy; NHI, national health insurance; LSEHS, late- stage elderly health insurance; OCS, oral corticosteroid.

Over the entire selection period, a confirmed diagnosis of MG was identified in 2,633 patients in the JMDC database, 2,787 patients in the NHI database and 3,201 patients in the LSEHS database. Of these, the general inclusion and exclusion criteria of the study were fulfilled for 251 patients in the JMDC population, 239 patients in the NHI population and 258 patients in the LSEHS population. After exclusion of patients with <2 years’ follow-up and those without an initial OCS treatment in the 90 days following the index date, the number of patients available for the present analysis was 161 for the JMDC, 150 for the NHI and 148 for the LSEHS (Figure 2).

### 3.2 Baseline characteristics

The characteristics of the patients are presented for the three populations in Table 1. There were more men than women in the JMDC and NHI populations, and more women than men in the LSEHS population. The mean age and the extent of comorbidity (proportion of patients with a CCI ≥ 1) was lowest in the JMDC population and highest in the LSEHS population. The proportion of patients with confirmed diabetes and osteoporotic fractures at inclusion was also highest in the LSEHS population.

**Table 1:** Baseline characteristics of the analysis population by OCS target achievement.

	All patients	≤5 mg OCS target achieved	≤5 mg OCS target not achieved	*p*-value*
JMDC population	*N* = 161	*N* = 94	*N* = 67	
Men, n (%)	94 (58.4%)	59 (62.8%)	35 (52.2%)	0.18
Age at index date (years), mean ± SD	48.5 ± 12.3	50.4 ± 12.2	45.9 ± 12.2	**0.02**
Hospital size (500 + beds), n (%)	113 (70.2%)	67 (71.3%)	46 (68.7%)	0.83
Charlson comorbidity index: 0 (low), n (%)	56 (34.8%)	33 (35.1%)	23 (34.3%)	0.99
Charlson comorbidity index: 1–2 (medium), n (%)	64 (39.8%)	37 (39.4%)	27 (40.3%)	
Charlson comorbidity index: ≥3 (high), n (%)	41 (25.5%)	24 (25.5%)	17 (25.4%)	
Confirmed diabetes before the index date, n (%)	8 (5.0%)	6 (6.4%)	2 (3.0%)	*0.47*
Osteoporotic fracture before the index date, n (%)	0 (0.0%)	0 (0.0%)	0 (0.0%)	NA
Visits for MG in the first 6 months of FU, mean ± SD	5.2 ± 2.4	4.8 ± 2.3	5.8 ± 2.3	**0.01**
NHI population	*N* = 150	*N* = 96	*N* = 54	
Men, n (%)	79 (52.7%)	50 (52.1%)	29 (53.7%)	0.85
Age at index date (years), mean ± SD	61.3 ± 10.0	62.4 ± 8.4	59.4 ± 12.3	0.08
Hospital size (500 + beds), n (%)	102 (68.0%)	66 (68.8%)	36 (66.7%)	0.79
Charlson comorbidity index: 0 (low), n (%)	49 (32.7%)	31 (32.3%)	18 (33.3%)	0.72
Charlson comorbidity index: 1–2 (medium), n (%)	65 (43.3%)	40 (41.7%)	25 (46.3%)	
Charlson comorbidity index: ≥3 (high), n (%)	36 (24.0%)	25 (26.0%)	11 (20.4%)	
Confirmed diabetes before the index date, n (%)	9 (6.0%)	8 (8.3%)	1 (1.9%)	*0.16*
Osteoporotic fracture before the index date, n (%)	1 (0.7%)	0 (0.0%)	1 (1.9%)	*>0.99*
Visits for MG in the first 6 months of FU, mean ± SD	5.2 ± 2.3	5.0 ± 2.4	5.6 ± 1.9	0.12
LSEHS population	*N* = 148	*N* = 119	*N* = 29	
Men, n (%)	60 (40.5%)	50 (42.0%)	10 (34.5%)	0.46
Age at index date (years), mean ± SD	79.8 ± 4.5	79.9 ± 4.6	79.6 ± 3.9	0.74
Hospital size (500 + beds), n (%)	96 (64.9%)	76 (63.9%)	20 (69.0%)	0.61
Charlson comorbidity index: 0 (low), n (%)	38 (25.7%)	30 (25.2%)	8 (27.6%)	0.66
Charlson comorbidity index: 1–2 (medium), n (%)	46 (31.1%)	39 (32.8%)	7 (24.1%)	
Charlson comorbidity index: ≥3 (high), n (%)	64 (43.2%)	50 (42.0%)	14 (48.3%)	
Confirmed diabetes before the index date, n (%)	14 (9.5%)	10 (8.4%)	4 (13.8%)	*0.48*
Osteoporotic fracture before the index date, n (%)	7 (4.7%)	2 (1.7%)	5 (17.2%)	***0.01***
Visits for MG in the first 6 months of FU, mean ± SD	5.6 ± 2.8	5.3 ± 2.8	6.8 ± 2.8	**<0.01**

NHI: national health insurance; LSEHS: late-stage elderly health insurance; OCS: oral corticosteroids; MG: myasthenia gravis; FU: follow-up; SD: standard deviation; NA: not assessable. *Values were compared between patients who achieved the OCS target dose and those who did not using the χ^2^ test or Fisher’s exact test (in italic) as appropriate for categorical variables and Student’s *t*-test or the Kruskal-Wallis test (in italic) as appropriate for continuous variables.*P* values in bold indicate significant between-group differences.

### 3.3 Achievement of the OCS target dose

Ninety-four patients in the JMDC population (58.4%), 96 patients in the NHI population (64.0%) and 119 patients in the LSEHS population (80.4%) achieved the target dose of ≤5 mg/day OCS during the two-year follow-up period (Figure 2).

**Figure 3:**
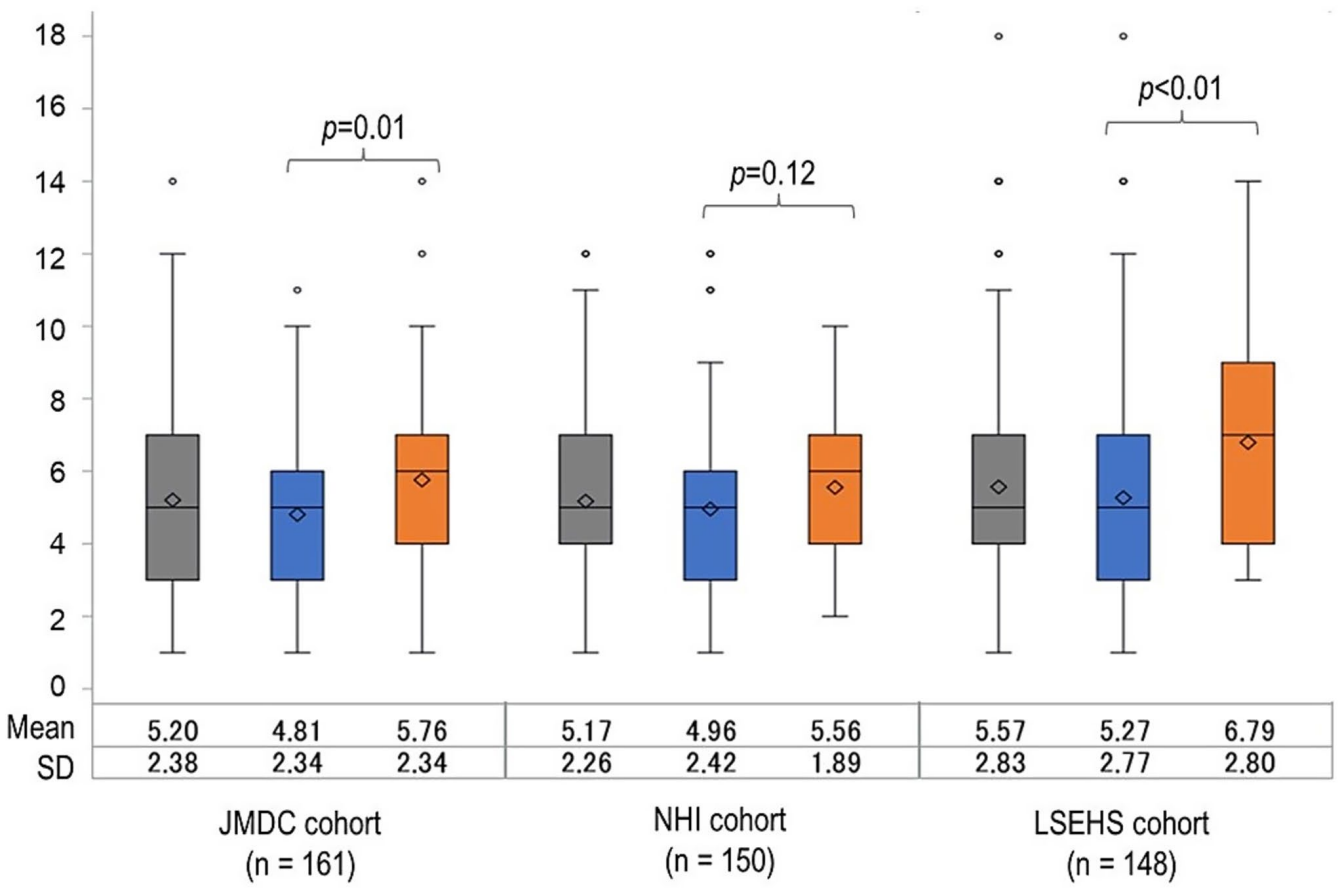
Distribution of number of visits for MG in the first 6 months of follow-up. NHI, national health insurance; LSEHS, late- stage elderly health insurance; OCS, oral corticosteroid. 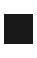, All patients; 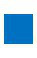, patients achieving the ≤5 mg/day OCS dose; 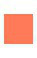, patients not achieving the ≤5 mg/day OCS dose.

In the JMDC population, patients who achieved the target were significantly older than those who did not (*p* = 0.02; Table 1). Otherwise, no significant difference between patients who achieved their OCS target and those that did not were observed for any of the other baseline characteristics in any of the three populations, with the exception of visits for MG (Figure 3). During the first 6 months of follow-up, patients who achieved their target made fewer visits for MG than those who did not in the JMDC population (*p* = 0.01) and the LSEHS population (*p* < 0.01), but not significantly in the NHI population (*p* = 0.12).

### 3.4 Incidence of adverse events of special interest

**Figure 4:**
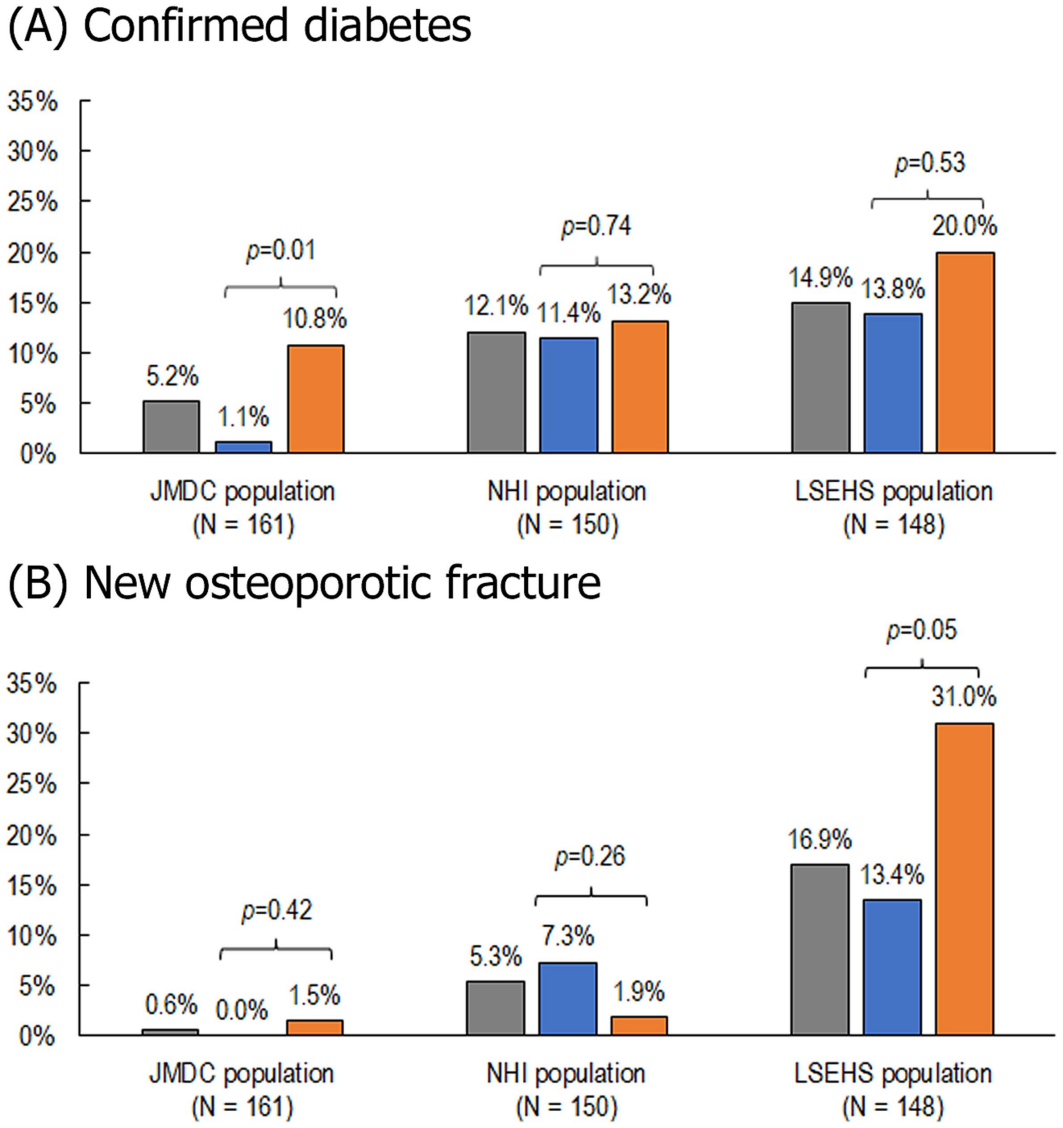
Incidence of confirmed diabetes and osteoporotic fractures. **(A)** Confirmed diabetes. **(B)** New osteoporotic fractures. NHI, national health insurance; LSEHS, late-stage elderly health insurance; OCS, oral corticosteroid. Patients with confirmed diabetes at baseline were excluded from this analysis. 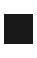, All patients; 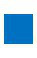, patients achieving the ≤5 mg/day OCS dose; 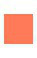, patients not achieving the ≤5 mg/day OCS dose.

New cases of confirmed diabetes over the two-year follow-up period were documented in 8 (5.2%) patients in the JMDC population, 17 (12.1%) of those in the NHI population and 20 (14.9%) of those in the LSEHS population (Figure 4A). In the JMDC population, seven of the eight patients who developed diabetes were patients who did not achieve the ≤5 mg/day OCS target (*p* = 0.01 versus achieving the target). No significant difference in the frequency of confirmed diabetes was observed in the other two populations. In the multivariate analysis, the association between achieving the ≤5 mg/day OCS target and new-onset confirmed diabetes in the JMDC population remained significant, with an odds ratio of 0.07 [95% CI, 0.01, 0.64] (*p* = 0.018; Table 2).

**Table 2:** Association between achievement of ≤5 mg/day OCS dose and adverse events.

	N	Yes	No	Univariate		Multivariate**	
				OR [95% CI]	*p*	OR [95% CI]	*p*
JMDC population							
Confirmed diabetes	152*	8	144	0.10 [0.01; 0.79]	**0.030**	0.07 [0.01; 0.64]	**0.018**
Osteoporotic fracture	*160*	*1*	*159*	NC	-	NC	-
NHI population							
Confirmed diabetes	141*	17	124	0.84 [0.30; 2.37]	0.745	0.73 [0.24; 2.25]	0.587
Osteoporotic fracture	150	8	142	4.17 [0.50; 34.82]	0.187	3.62 [0.42; 31.16]	0.242
LSEHS population							
Confirmed diabetes	134*	20	114	0.64 [0.21; 1.96]	0.433	0.74 [0.22; 2.42]	0.616
Osteoporotic fracture	148	25	123	0.35 [0.13; 0.89]	**0.028**	0.36 [0.13; 1.01]	0.052

NHI: national health insurance; LSEHS: late-stage elderly health insurance; OCS: oral corticosteroid. OR: odds ratio; CI: confidence intervals. *Patients with confirmed diabetes at baseline were excluded from these analyses.**Model adjusted to age at the index date (years), sex, hospital size (500 + vs. < 500 beds) and number of MG visits in the first 6 months of follow-up.*P* values in bold indicate significant between-group differences.

New osteoporotic fractures occurring during the two-year follow-up period were documented in 1 (0.6%) patient in the JMDC population, 8 (5.3%) of those in the NHI population and 25 (16.9%) of those in the LSEHS population (Figure 4B). In the LSEHS population, the frequency of new osteoporotic fractures was significantly higher (*p* = 0.049) in patients who did not achieve the target than in those who achieved it. However, this association was not retained in the multivariate analysis (*p* = 0.052, Table 2). No difference in the frequency of new osteoporotic fractures was observed between achievers and non-achievers in the JMDC and NHI populations.

### 3.5 Costs

Median annualized MG-related medical costs and total medical costs over the 2 years of follow-up were significantly higher in patients who did not achieve the ≤5 mg/day OCS target than in those who achieved the target in both the JMDC population (*p* < 0.01 for MG-related costs and *p* = 0.01 for total costs) and the LSEHS population (*p* < 0.01 for both MG-related and total costs; Table 3). Smaller differences were observed in the NHI population, which did not reach statistical significance (*p* = 0.16 for MG-related costs and *p* = 0.37 for total costs).

**Table 3:** Total and myasthenia-gravis-related medical costs.

	All patients	≤5 mg OCS target achieved	≤5 mg OCS target not achieved	*p*-value*
JMDC population	*N* = 161	*N* = 94	*N* = 67	
*Median annualized costs over the 2 years of follow-up* [*yens × 1,000*]				
MG-related costs	796 [274; 1,511]	581 [215; 1,139]	1,147 [370; 2,335]	**<0.01**
Total medical costs	878 [410; 1,667]	772 [391; 1,316]	1,169 [585; 2,346]	**0.01**
*Change in annualized costs between the baseline period and the first 2 years of follow-up* (*%*)				
MG-related costs	172.7 [−36.2; 943.4]	137.0 [−26.5; 1,006.3]	212.2 [−41.0; 899.2]	0.65
Total medical costs	63.9 [−39.0; 633.4]	58.9 [−39.0; 387.5]	90.3 [−42.6; 692.4]	0.89
*Median annualized costs over the 2 years of follow-up* [*yens × 1,000*]				
MG-related costs	756 [244; 1,612]	699 [243; 1,481]	943 [246; 2,245]	0.16
Total medical costs	923 [456; 1,916]	892 [469; 1,513]	1,000 [430; 2,351]	0.37
*Change in annualized costs between the baseline period and the first 2 years of follow-up* (*%*)				
MG-related costs	168.3 [−40.0; 802.3]	133.0 [−48.4; 793.5]	261.7 [−24.1; 1,001.0]	0.11
Total medical costs	147.2 [−34.5; 652.8]	133.2 [−40.5; 560.1]	156.7 [−19.4; 934.2]	0.15
LSEHS population, n	*N* = 148	*N* = 119	*N* = 29	
*Median annualized costs over the 2 years of follow-up* [*yens × 1,000*]				
MG-related costs	757 [286; 1,518]	605 [238; 1,213]	1,638 [687; 2,496]	**<0.01**
Total medical costs	1,132 [686; 1,974]	1,035 [627; 1,686]	2,284 [1,055; 3,051]	**<0.01**
*Change in annualized costs between the baseline period and the first 2 years of follow-up* (*%*)				
MG-related costs	318.4 [−3.4; 846.4]	315.9 [4.6; 773.0]	498.3 [−43.2; 1,734.8]	0.39
Total medical costs	257.4 [20.7; 791.2]	225.3 [40.6; 685.2]	292.3 [−12.6; 872.4]	0.69

NHI, national health insurance; LSEHS, late-stage elderly health insurance. MG, myasthenia gravis. All data are presented as median values with the first and the third quartiles.*Values in the two subgroups of patients achieving and not achieving their OCS dose target were compared with the Kruskal-Wallis test.*P* values in bold indicate significant between-group differences.

## 4 Discussion

In this retrospective study, we observed that OCS-related complications (diabetes and osteoporotic fractures) were more frequent in patients who did not achieve the ≤5 mg/day OCS target than in those who achieved the target. In addition, total and MG-related costs, were higher in patients who did not achieve the target. These associations were not always statistically significant, which may reflect the fact that MG is a rare disease and absolute patient numbers were low. It may also reflect characteristics of insurance systems between the three populations.

In the JMDC population, seven of the eight patients who developed diabetes during the two-year follow-up period did not achieve the ≤5 mg/day OCS target, corresponding to an OR of 0.07 for patients who achieved the treatment target versus those who did not. However, this increase in the risk of diabetes was only observed in the JMDC population. This could possibly be explained by the younger age of these patients (mean: 48.5 years) and the relatively low frequency of comorbid diabetes at baseline (7.5%). In the NHI and LSEHS populations, with a mean age of 61 years and 80 years respectively, the frequency of diabetes at baseline was >13%. Since the age of onset of type 2 diabetes in Japan is typically over 60 years (28), the additional risk of diabetes associated with high-dose OCS exposure may be masked by other risk factors in the older patients in the NHI and LSEHS populations, such as low bone density, frailty, limited mobility and age-related comorbidities such as dementia, Parkinson’s disease or chronic kidney disease (26, 29, 30). These observations align with a recent study in MG patients, which confirmed that corticosteroid treatment significantly increases the risk of diabetes, while suggesting that the magnitude of this effect may vary according to patient age and baseline metabolic risk (31).

In contrast, the excess risk of osteoporotic fractures in patients who did not achieve the OCS dose target was only observed in the LSEHS population (13.4% in patients who achieved the target and 31.0% in those who did not, OR, 0.36). This population was the oldest, representing individuals aged over 75 years with a mean age of 80 years. Given that old age is a major bone fragility risk factor (32), it is possible that older patients are more vulnerable to the deleterious effect of OCS on bone integrity. Recent studies reported higher risk of osteoporosis associated with OCS use in patients with COPD and asthma with a clear cumulative OCS dose effect among OCS users (33–35). Fracture risk increases rapidly after initiation of OCS treatment and is strongly dependent on dose and treatment duration (36). There are less data available in patients with MG and there is no clear consensus on the findings (7, 8, 37). A number of studies from Asia have reported an increased risk of osteoporosis or osteoporotic fracture in patients with MG compared to controls, as well as an association with OCS exposure (7). In contrast, studies in Europe or Canada have failed to demonstrate a significant association (38–40). Given that patients with MG in Japan are frequently treated with higher-dose OCS for prolonged periods of time, further studies on this association between cumulative exposure to OCS, age and osteoporotic fracture in patients with MG are clearly merited.

The age-related differences in OCS complications may also be explained by differences in the underlying pathophysiological mechanisms of these conditions. Glucocorticoid-induced diabetes can occur relatively quickly, especially in younger individuals with fewer comorbidities (13), whereas osteoporosis develops gradually and is more pronounced in older adults due to age-related bone loss (36).

Across all three populations, MG-related medical costs were substantial and accounted for the majority of total medical costs. These costs were consistently higher among patients who did not achieve the ≤5 mg/day OCS. As MG-related costs dominated total costs, overall expenditures were also higher in these patients. This may reflect more frequent or severe disease activity among those not achieving the target.

The strengths of the study include the evaluation of patients with MG in three databases, which provided fairly consistent findings between populations. Given that MG is a rare disease, the use of multiple sources enabled enrolment of larger numbers of patients than would have been possible with a single data source, and as a result, this is one of the largest health insurance database studies on MG patients in Japan. In addition, the same coding conventions are used in three databases which ensures that data are comparable between sources. The limitations include the absence of information on disease phenotype (antibody status), clinical manifestations, functional impairment and severity. Notably, patients diagnosed with ocular MG cannot be distinguished from those with generalized MG based on ICD-10 codes, and OCS use may differ between these two groups. Similarly, information is missing on severity, which may be a determinant of both OCS use and outcomes. However, we considered the number of MG-related visits during the first 6 months of follow-up as a potential proxy for disease severity and included it as a key covariate in our analyses. Interestingly, the number of MG visits was higher in patients who did not achieve the ≤5 mg/day OCS, which may reflect that patients with higher disease activity had more difficulties with the achievement of the OCS dose target. Furthermore, documentation of OCS was based on dispensing in claims data, and no information is available on actual adherence. Another limitation is that causality between OCS prescription and adverse events cannot be assessed in a cross-sectional analysis (41). However, it was possible to demonstrate the disease burden associated with different treatment patterns. Finally, the validity of diagnoses of diseases in health insurance claims databases may be limited as they are registered for reimbursement purposes. To optimize case ascertainment, we defined cases using both disease codes and specific medication codes.

It should also be noted that we did not distinguish between patients achieving the target who continued to receive low-dose OCS and those discontinuing OCS completely. It is possible that outcomes in these two groups are not the same.

Other types of study, such as observational studies in the Japan MG registry (JAMG-R), or surveys of patients or physicians, may help characterize the impact of reduction of long-term exposure to higher-dose OCS on patients’ well-being. Such studies would help to determine whether the overall treatment target of the 2014 Japanese clinical practice guidelines for MG (15, 16) to improve patients’ QoL is being achieved (42).

In conclusion, patients who did not achieve the target of ≤5 mg/day OCS carry a higher burden than those who did achieve this target, in terms of increased steroid-related complications (diabetes and osteoporotic fractures) and a high cost of total and MG-related medical care. More widespread implementation of effective treatment strategies is required to reduce long-term use of higher-dose OCS and the associated economic burden (43).

## 5 Conclusion

Patients who do not achieve the target of ≤5 mg/day OCS carry a higher burden than those who do achieve this target, in terms of increased steroid-related complications (diabetes and osteoporotic fractures) and a high cost of total and MG-related medical care. Broad adoption of effective treatment approaches is essential to minimize prolonged reliance on higher-dose OCS and alleviate the related economic impact on patients with MG.

## Data Availability

The data analyzed in this study is subject to the following licenses/restrictions: the data that support the findings of this study are available from JMDC Inc. and DeSC Healthcare Inc., but restrictions apply to their availability, which were used under license for the current study, and so are not publicly available. Reasonable requests for access to the data can be addressed to the authors who will transfer them to JMDC or DeSC. Requests to access these datasets should be directed to Yohei Ohashi, Yohei.Ohashi@ucb.com.
